# The Effect of Silencing the Genes Responsible for the Level of Sphingosine-1-phosphate on the Apoptosis of Colon Cancer Cells

**DOI:** 10.3390/ijms24087197

**Published:** 2023-04-13

**Authors:** Adam R. Markowski, Arkadiusz Żbikowski, Piotr Zabielski, Urszula Chlabicz, Patrycja Sadowska, Karolina Pogodzińska, Agnieszka U. Błachnio-Zabielska

**Affiliations:** 1 Department of Internal Medicine and Gastroenterology, Polish Red Cross Memorial Municipal Hospital, 79 Henryk Sienkiewicz Street, 15-003 Bialystok, Poland; 2 Department of Medical Biology, Medical University of Bialystok, 2C Adam Mickiewicz Street, 15-222 Bialystok, Poland; 3 Department of Hygiene, Epidemiology and Metabolic Disorders, Medical University of Bialystok, 2C Adam Mickiewicz Street, 15-222 Bialystok, Poland

**Keywords:** gene silencing, sphingosine-1-phosphate, ceramides, cellular apoptosis, colon cancer cells

## Abstract

Sphingosine-1-phosphate (S1P) and ceramides (Cer) are engaged in key events of signal transduction, but their involvement in the pathogenesis of colorectal cancer is not conclusive. The aim of our study was to investigate how the modulation of sphingolipid metabolism through the silencing of the genes involved in the formation (*SPHK1*) and degradation (*SGPL1*) of sphingosine-1-phosphate would affect the sphingolipid profile and apoptosis of HCT-116 human colorectal cancer cells. Silencing of *SPHK1* expression decreased S1P content in HCT-116 cells, which was accompanied by an elevation in sphingosine, C18:0-Cer, and C18:1-Cer, increase in the expression and activation of Caspase-3 and -9, and augmentation of apoptosis. Interestingly, silencing of *SGLP1* expression increased cellular content of both the S1P and Cer (C16:0-; C18:0-; C18:1-; C20:0-; and C22:0-Cer), yet inhibited activation of Caspase-3 and upregulated protein expression of Cathepsin-D. The above findings suggest that modulation of the S1P level and S1P/Cer ratio regulates both cellular apoptosis and CRC metastasis through Cathepsin-D modulation. The cellular ratio of S1P/Cer seems to be a crucial component of the above mechanism.

## 1. Introduction

Colorectal cancer (CRC) is one of the most commonly diagnosed cancers worldwide and therefore is of interest to many research teams [1]. Most cases of colorectal cancer are thought to develop through the accumulation of molecular and genetic alterations [2], including activation of specific oncogenes, inactivation of tumor suppressors, and additional epigenetic changes [3]. The CRC incidence trends between age groups are different, and a particularly worrying change is observed among younger people. Recently, it was established that the incidence rate of CRC for individuals aged 50 to 64 years increases by 1% per year, whereas for those under 50 years old it increases by 2% per year [4]. In order to find the cause of this phenomenon, it is necessary to recognize and understand as many aspects of the metabolism of cancer cells as possible.

**Figure 1:**
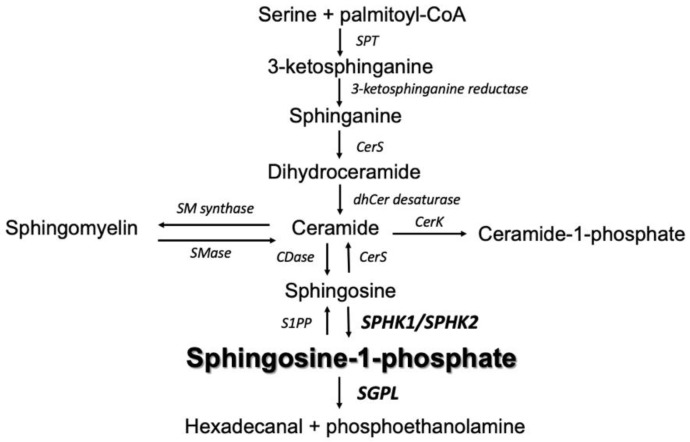
Overview of sphingolipid metabolism. SPT serine palmitoyltransferase. CerS ceramide synthase. CerK ceramide kinase. dhCer desaturase dihydroceramide desaturase. CDase ceramidase. SMase sphingomyelinase. SM synthase sphingomyelinase synthase. S1PP sphingosine-1-phosphate phosphatase. SPHK1/SPHK2 sphingosine kinase 1 and 2, respectively. SGPL sphingosine 1-phosphate lyase.

Sphingolipids are a bioactive lipid group that participates in many crucial cellular functions (Figure 1). These compounds are involved in key events of signal transduction and cell regulation [5], such as cell adhesion and migration, aging, inflammatory response, and also in processes of proliferation, differentiation, growth arrest, and apoptosis. The literature data indicate that the cellular levels of sphingosine-1-phosphate (S1P) and ceramides (Cer) play a significant role in the pathogenesis of CRC, and the S1P/Cer ratio (described as sphingolipid rheostat) decides whether the cells will be directed towards proliferation or cellular senescence and apoptosis [6,7]. It should be emphasized that Cer and S1P play opposite roles in oncogenesis; ceramide is a pro-apoptotic compound, while S1P is a pro-proliferative compound. Hence, deregulation of sphingolipid metabolism in colorectal cancer cells, resulting in an increase in S1P levels, may contribute to their excessive proliferation and malignant transformation [8]. Moreover, extracellular S1P affects cellular motility and was shown to stimulate migration, sphere-forming ability, and stemness of CRC cells, leading to metastasis [9]. The results of lipidomic studies confirmed that, during the experimentally induced apoptosis in CRC cell lines, the greatest quantitative changes in the lipid profile concerned sphingolipids [10]. However, there is little data on the real role of ceramides in the pathogenesis of CRC, and existing data on this topic are not conclusive. Some studies have shown that the content of ceramide in human colorectal cancer samples was significantly lower compared to the normal colon mucosa [11]. On the other hand, there are data indicating that the total content of ceramides in tumor tissue was higher than in adjacent non-tumor tissue [1].

Studies performed on animal models and in vitro on human colon cancer cells have confirmed that many enzymes involved in sphingolipid metabolism are dysregulated in cancer cells. Due to the pro-proliferative properties of S1P, sphingosine kinases 1 and 2 (SPHK1 and SPHK2) received significant attention. SPHK1 is mainly localized in cytoplasm and plasma membrane and releases S1P both intra- and extracellularly, whereas SPHK2 localizes in the nucleus, mitochondria, and endoplasmic reticulum and produces intracellular S1P [12]. Although both isoforms seems to play a role in the regulation of cell cycle, the data show that SPHK2 can both stimulate or suppress cellular proliferation depending on cell type or expression level. In several studies, SPHK2 displayed pro-apoptotic properties inducing cell death in various cell lines [13,14,15]. In some cellular models, SPHK2 expression was crucial for the apoptotic signaling [16]. Involvement of SPHK2 in inhibition of cellular death seems to be connected with nuclear translocation and generation of S1P at the level of nuclei. Nevertheless, SPHK2 was shown to promote CRC proliferation [17], confer the resistance to 5-fluorouracil [18], and increase survivability of CRC cells in serum-deprived conditions [19]. Also, targeting of SPHK2 through shRNA or isoform-specific inhibitors was shown to inhibit cellular proliferation [20], and increase susceptibility of CRC to all-trans retinoic acid [21] or oxaliplatin treatment [22]. SPHK1 involvement in the stimulation of cellular proliferation is better defined [23]. Its expression has been shown to be significantly higher in human colon adenocarcinomas than in the normal surrounding epithelium, and simultaneously higher in metastatic CRC tissue than in non-metastatic CRC [24,25]. Thus, suppression of S1P synthesis trough SPHK1 targeting seems to be a more attractive approach compared to SPHK2 modulation in CRC cells. Other studies in CRC demonstrated decreased activity of enzymes responsible for cellular reduction of S1P levels through its dephosphorylation (by S1P phosphatase—S1PP) or irreversible degradation (by S1P lyase—SGPL) [26]. Although the activity of both enzymes decreases cellular S1P content, their effect on sphingolipid metabolism differs significantly. S1P dephosphorylation through SPP1 increases cellular sphingosine, and subsequently, ceramide levels in a similar fashion to SPHK inhibition, and stimulates cellular apoptosis [27], whereas inhibition of SGPL1 activity blocks the pathway of permanent removal of bioactive sphingoid backbones, which can trigger apoptosis [28,29]. In addition, it has been observed that the activity of alkaline sphingomyelinase, the enzyme responsible for the hydrolysis of sphingomyelin to ceramide, is significantly decreased in human colorectal carcinoma [30]. Furthermore, it has been found that inhibition of neutral ceramidase in colon cancer cells increases ceramide levels, and this is accompanied by a decrease in proliferation and cell survival and an increase in apoptosis [31]. The above data reinforce the statement that changes in sphingolipid metabolism may play an important role in the development and progression of colorectal cancer. Cancer arises as a consequence of an imbalance between cell proliferation and cell death; malfunctioning signaling pathways allow healthy cells to transform into cancer cells. Apoptosis is a naturally occurring, tightly regulated process through which damaged or unwanted cells are removed from an organism to make space for new tissues and maintain cellular homeostasis [32]. The literature data have shown that sphingolipids trigger apoptotic or proliferative signals in cancer cells through several possible mechanisms, including activation of RAS/MEK/ERK and PI3K/AKT pro-survival pathways (S1P, through G-protein-coupled S1PR receptors), stimulation of TNFα signaling (aSMase-mediated Cer release), or induction of mitochondrial depolarization and cytochrome-C release (SPH and/or Cer through direct effects) [12,33]. One of the proposed mechanisms is the activation and inhibition of caspases [34,35,36,37]. Caspases are synthesized as inactive forms, and under external or internal stimuli they activate each other in a reaction cascade [38]. When activated, initiator caspases (Caspase-2, -8, -9, -10) launch executioner caspases (Caspase-3, -6, -7), which lead to cleaving key structural and regulatory proteins to dismantle the cell from within [39]. However, cancer cells often avoid apoptosis, being able to promote their own growth and prevent removal, by blocking dimerization of initiator caspases, producing caspase inhibitors, and receiving mitogenic signals for the proliferation from neighboring cells; deregulation of caspase expression and activation contributes to cancer development [40,41]. However, tumor formation requires not only cell proliferation but also extracellular matrix remodeling. Cathepsins are well-known pro-invasive enzymes that, by degradation of proteins of the extracellular matrix as well as by processing of various growth factors or cytokines, remodel the tumor microenvironment and contribute to their growth, invasion, and metastasis; Cathepsin-D (CTSD) is the major lysosomal protease in mammals [42]. However, the role of Cathepsin-D in cell death appears to be the multifaceted, depending on its localization, condition of cells, and context in cancer cells [42]. This enzyme is mainly found in lysosomes, but under certain conditions, it can also be localized in the cytosol and extracellular space, which may explain its different abilities to modulate cell death. Cathepsin-D activity increases uncontrolled in cancer cells and its high extracellular level has been detected also in colorectal cancer [43]. This enzyme can break intercellular junctions, causing cancer cells to detach from one another and allow them to migrate through the body, leading to metastasis. Sphingolipids were shown to act as both the activators and inhibitors of Cathepsin-D, with ceramide-1-phosphate being able to activate, and sphingosine and short-chain ceramide inhibit, its activity by direct in situ molecular binding [44].

Also, S1P was shown to regulate proliferation through the impact on caspases and cathepsins, including Cathepsin-D [45,46]. Moreover, in addition to direct effects, sphingolipids are able to indirectly affect lysosomal/endosomal release and cellular localization of Cathepsin-D. The literature data indicate that aSMase-mediated endosomal ceramide accumulation is crucial for the endosomal release of Cathepsin-D, which subsequently triggers Caspase-3 and -9 activation and apoptosis in TNFα-treated cells [47]. Similarly, sphingosine accumulation is crucial for lysosomal permeabilization and subsequent release and activation of cathepsins [48,49]. Both processes release cathepsins intracellularly, aiding in caspase-mediated cell death. Much less is known about the effects of S1P on the activity/expression of Cathepsin-D, yet the S1P-mediated upregulation of extracellular Cathepsins-D could be one of the factors in CRC metastasis.

S1P possesses strong pro-proliferative properties, but data on the role of S1P in carcinogenesis are not conclusive. Therefore, the aim of the study was to investigate how the modification of sphingolipid metabolism through silencing of genes involved in the formation (*SPHK1*) and degradation (*SGPL1*) of sphingosine-1-phosphate change the S1P/Cer rheostat and affect the proliferation and apoptosis of CRC cells. To answer this question, we measured the cellular content of both ceramides and S1P, along with the expression of proteins involved in the proliferation/apoptosis pathways, and assessed the apoptosis rate of colon cancer cells in which the genes encoding SPHK1 and SGPL1 had been silenced.

## 2. Results

### 2.1. Gene Silencing

**Figure 2:**
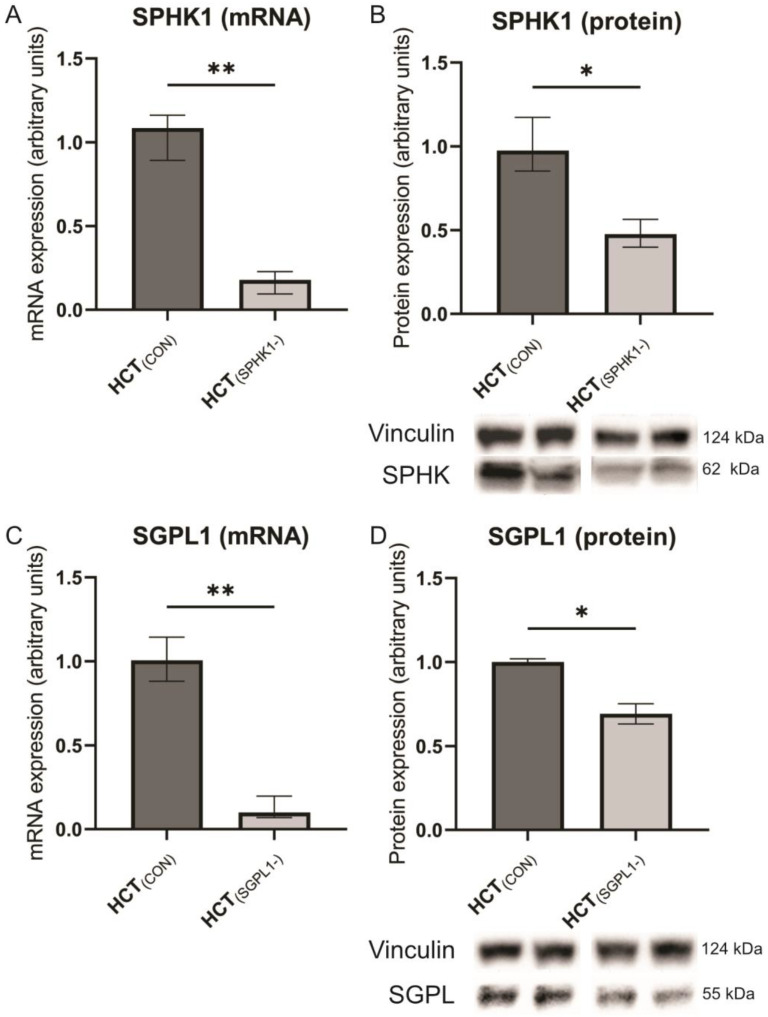
The impact of particular gene silencing on the expression of SPHK1 (panels **A** and **B**) and SGPL1 (panels **C** and **D**) in human colorectal carcinoma HCT-116 cell line. Values are control-normalized medians +/− interquartile ranges (n = 6 qPCR; n = 4 WB). Bands from two independent representative WBs are presented below protein expression graphs; * *p* < 0.05; ** *p* < 0.01 vs. control (HCT_(CON)_).

Both the RT-PCR assay and WB confirmed a significant reduction of *SPHK1* mRNA and protein expression in silenced cells as compared to non-silenced control cells (*p* = 0.0022 and *p* = 0.0286, respectively) (Figure 2A,B). A similar profile of changes was observed in *SGPL1*-silenced cells at the level of mRNA and protein (*p* = 0.0022 and *p* = 0.0286, respectively) (Figure 2C,D).

### 2.2. Sphingolipids Content

**Figure 3:**
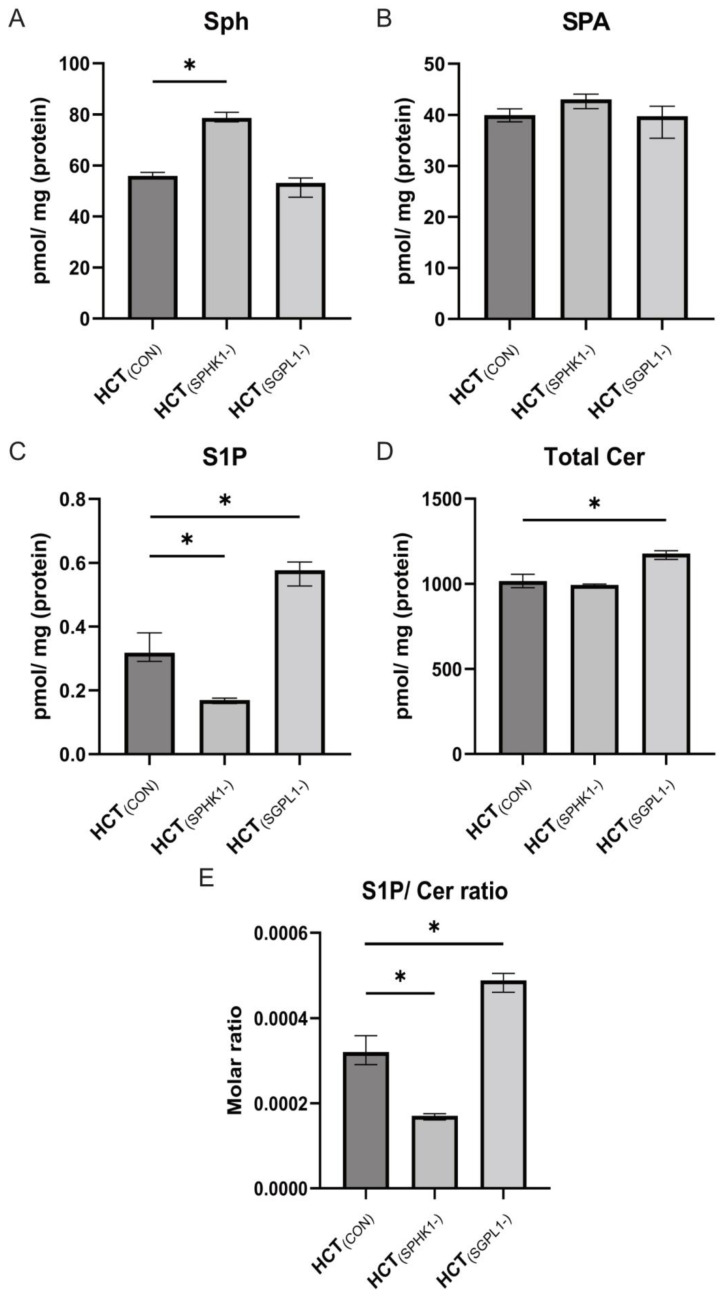
The effect of *SPHK1* and *SGPL1* silencing on the content of free sphingoid bases (Sph, SPA; panels **A** and **B**), sphingosine-1-phosphate (S1P, panel **C**), total ceramide (panel **D**) and S1P/Cer ratio (panel **E**) in human colorectal carcinoma HCT-116 cells. The S1P/Cer ratio was normalized to control. Values are medians +/− interquartile ranges (n = 4); * *p* < 0.05; vs. control (HCT_(CON)_).

The silencing of genes encoding enzymes directly responsible for the level of S1P caused significant changes in the content of not only S1P, but also other sphingolipids. In cells in which the expression of *SPHK1* was silenced, a significant increase in the content of sphingosine (Sph) (*p* = 0.0286) and a decrease in the content of S1P (*p* = 0.0286) were observed compared to control cells (Figure 3A,C). Apart from that, an increased content of C18:0-Cer and C18:1-Cer was observed in relation to the non-silenced control cells (*p* = 0.0286) (Table 1). A significant reduction in the S1P/Cer ratio (*p* = 0.0286) was also observed (Figure 3E).

In the case of silencing the *SGPL1* gene, significant changes in the content of sphingolipids were also found compared to the control, non-silenced cells. Although the Sph and SPA levels did not change, there was an increase in the content of S1P, 16:0-Cer, C18:0-Cer, C18:1-Cer, C20:0-Cer, C22:0-Cer, and the total content of ceramides (*p* = 0.0286) (Table 1). Moreover, an increase in the S1P/Cer ratio was observed (*p* = 0.0286) (Figure 3E).

### 2.3. Caspases and Cathepsin-D

**Figure 4:**
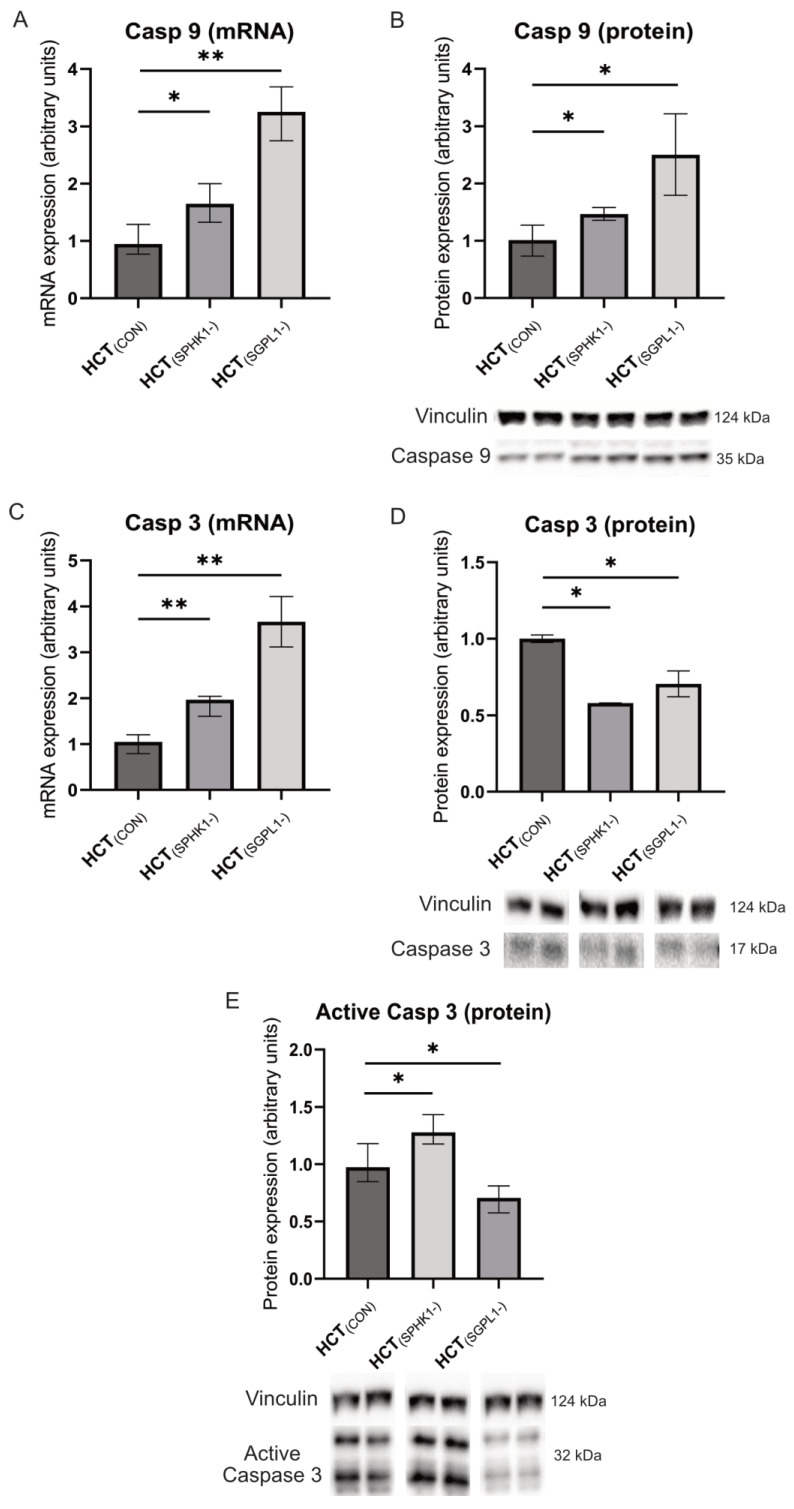

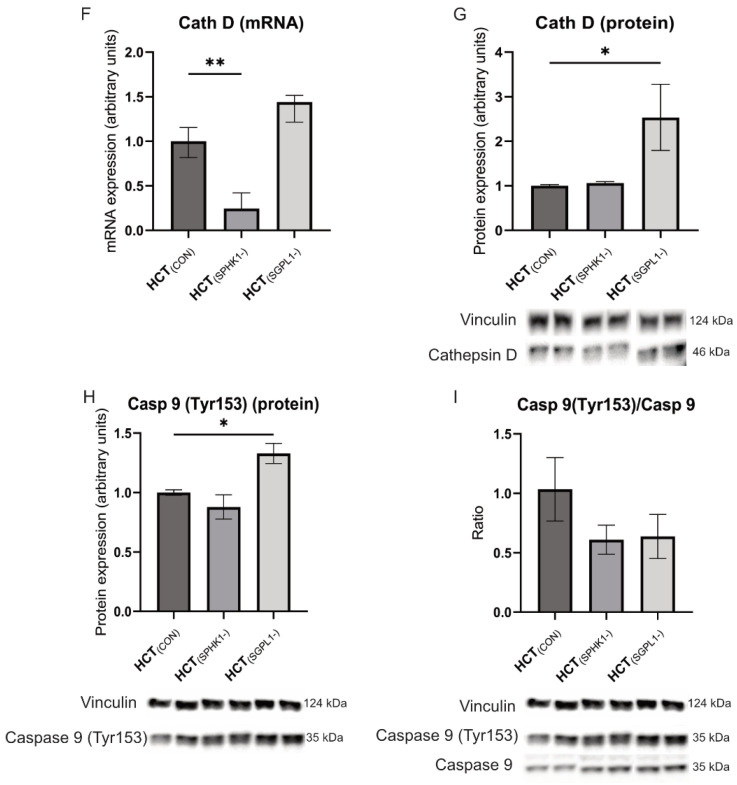
The impact of *SPHK1* and *SGPL1* gene silencing in human colorectal tumor HCT-116 cell line on the expression and phosphorylation state of initiator Caspase-9 (panels **A**,**B**,**H**,**I**), executioner Caspase-3 (panels **C**–**E**) and Cathepsin-D (panels **F**,**G**). Two lower bands in Panel 4E show active (cleaved) Caspase-3. Protein bands from two independent representative WBs are presented below expression protein graphs. Values are control-normalized medians +/− interquartile ranges (n = 6 qPCR; n = 4 WB). * *p* < 0.05; ** *p* < 0.01 vs. control (HCT_(CON)_).

The RT-PCR technique revealed that silencing the *SPHK1* gene in HCT-116 cells resulted in an increase in the expression level of caspases (Caspase-9 and Caspase-3) (*p* = 0.0152 and *p* = 0.0087, respectively) (Figure 4A,C) and a decrease in Cathepsin-D expression at the mRNA level (*p* = 0.0022) (Figure 4F). Western blot analysis showed an increased level and activation of Caspase-3 (Figure 4D,E), and an increased level of Caspase-9 compared to control, non-silenced cells (*p* = 0.0286) (Figure 4B). In addition, we noticed a negative trend in phosphorylation of the Tyr153 tyrosine residue in the Caspase-9 protein compared to non-silenced control cells (Figure 4H).

Silencing of the *SGPL1* gene in HCT-116 cells upregulated Caspase-9, Caspase-3, and Cathepsin-D at the mRNA level as compared to non-silenced cells (Figure 4A,C,F). At the protein level, we observed a decreased Caspase-3 content and activation state (*p* = 0.0286) (Figure 4D,E) and an increased Caspase-9 and Cathepsin-D content as compared to non-silenced control cells (*p* = 0.0286) (Figure 4B,G). Moreover, despite the high content of Caspase-9 and its Tyr153-phosphorylated form, which suggest its activation, we observed a negative trend in the phosphorylation state of this protein in *SGPL1*-silenced cells as compared to non-silenced control cells (Figure 4I).

### 2.4. Cellular Apoptosis

**Figure 5:**
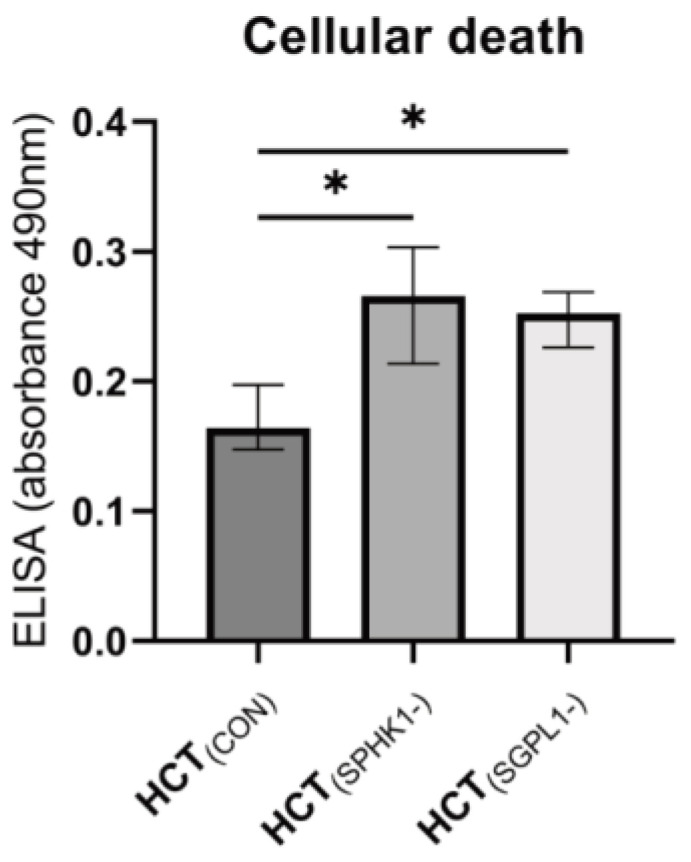
Cellular death by apoptosis (as measured by ELISA apoptosis assay) in *SPHK1*- and *SGPL1*-silenced human colon tumor cells. Values are medians +/− interquartile ranges (n = 4); * *p* < 0.05 vs. control (HCT_(CON)_).

The apoptosis test (ELISA PLUS) confirmed the increase in the death rate of both the *SPHK1* (*p* = 0.0286)- and *SGPL1* (*p* = 0.0286)-silenced cells (Figure 5).

## 3. Discussion

Sphingosine kinase performs an essential role in the regulation of the metabolic balance of sphingolipids such as ceramides and S1P. Ceramides inhibit cell proliferation and stimulate apoptosis, whereas S1P promotes cellular proliferation, motility, and survival. The content of these compounds in a cell is dynamically maintained, and the balance between them seems to act as a switch that determines the fate of the cell, choosing between cellular proliferation and death [50,51].

SPHK1 is located predominantly in the cytosol, but after exposure to appropriate stimuli, is activated by phosphorylation and translocated to the cell membrane. Active, membrane-bound sphingosine kinase mediates the conversion of sphingosine to sphingosine-1-phosphate (S1P). S1P acts as a secondary signal transmitter, taking part in apoptosis, proliferation, migration, and cancer invasion.

In our study, silencing the gene encoding SPHK1, the enzyme responsible for the formation of S1P, led to the expected decrease in S1P levels and caused a reduction in the ratio of sphingosine-1-phosphate to total ceramide content. The decrease in the level of S1P and the ratio of S1P to ceramide at the same time resulted in a significant increase in apoptosis in these cells. Our findings have clinical relevance, as they strongly confirm the cause and effect relationship between a low level of S1P and intensification of apoptosis in CRC cells.

Our conclusions are confirmed by the existing literature data. It has been shown that SPHK1 is upregulated in multiple types of human cancers [52], and the expression of SPHK1 in CRC tissue is significantly increased compared to normal colorectal tissue [53]. Elevated SPHK1 augments colon cancer cell proliferation [54], and SPHK1 overexpression in intestinal epithelium significantly increases tumor multiplicity [55]. On the other hand, the intestinal epithelial deletion of SPHK1 prevents colitis-associated cancer development in mice [56], SPHK inhibitor treatment resulted in a dose-dependent decrease in colitis-driven colon cancer in mice [57], and SPHK1 knockout mice demonstrated a lower incidence of colon cancer development in murine model [24].

It seems that SPHK1 regulates tumorigenesis and tumor growth in early colon cancer [55]. Moreover, SPHK1 induces the first step of a metastatic cascade, which is the epithelial-to-mesenchymal transition [58]. The positive SPHK1 expression in advanced colorectal cancer (stages III and IV) was higher than in less advanced tumors [58]. The expression density of SPHK1 in primary colorectal cancer tissues was higher compared with normal colonic mucosa tissues, whereas the expression of E-cadherin was lower [58]. Moreover, patients with SPHK1-positive colorectal cancer cells had a significantly lower survival rate compared with patients with SPHK1-negative cancer [58]. Additionally, the recent meta-analysis involving 32 cohorts with 5965 patients confirmed that high SPHK1 expression was significantly associated with poor overall survival and worse disease-free survival [59]. S1P is catabolized by SGPL1 and the S1P phosphatases. Since only SGPL1 irreversibly degrades S1P, it has a particularly strong influence on intracellular and tissue levels of S1P. S1P stimulates cell proliferation and promotes invasion in colon cancer cell lines [60], and neutralizing S1P with highly specific anti-S1P monoclonal antibodies blocks S1P’s pro-survival actions (by increasing activation of Caspase-3), which was manifested by the inhibition of tumor progression and reduction of the tumor volume in human cancer models [61]. In turn, S1P treatment decreased Caspase-3 protein levels and activity in nontumorigenic intestinal epithelial cells [62].

Caspases (a family of protease enzymes) are important mediators of apoptosis. There are two main signaling pathways involved in apoptosis: the extrinsic (death receptor) pathway and the intrinsic (mitochondrial) pathway. Both require Caspase-8 and Caspase-9 activation, respectively, and both activate Caspase-3, leading to cellular death. Caspase-3 translocates to the nucleus during apoptosis to cleave targets such as poly-ADP ribose polymerase [63]. In colon cancer cells with deficiency in CerS6 expression, Caspase-3 may, despite its activation, not be able to translocate to the nucleus, altering late-stage apoptotic signaling [64]. Caspase-3 regulates the migration, invasion, and metastasis of colon cancer cells. It has been proven that Caspase-3 knockout HCT-116 cells show altered cellular morphology and reduced facility to epithelial-to-mesenchymal transition [65]. It was recently shown that mRNA expressions of Caspase-9 and Caspase-3 (unlike Caspase-8) were downregulated in human colorectal cancer tissues in comparison to marginal tissues [66]. The same study also identified a correlation between the caspases expression and the severity of tumor stage, distant metastases, and differentiation stage, while no significant association was seen with age, sex, and smoking. Another study revealed a positive association of Caspase-3 expression with a patient’s age and tumor histological type, and a negative correlation with depth of tumor invasion, tumor grade, tumor stage (II to IV), and the presence of lymph node metastases [67]. On the other hand, no relationship was demonstrated between Caspase-3 expression and patients’ sex, tumor location (right colon vs. left colon), tumor size, and presence of lymphovascular invasion or perineural invasion [67]. The above findings underline the diversity of the Caspase function in CRC, which can arise from diversity of molecular pathways responsible for carcinogenesis.

In our study, we have noticed an increase in the expression of Caspase-9 and Caspase-3 at the mRNA level after *SPHK1* gene silencing. However, at the protein level, we observed only an increase in the Caspase-9 protein expression, while the Caspase-3 protein was decreased. Despite this, Caspase-3 active form (cut into low molecular mass subunits) displayed upregulation in *SPHK1*-silenced cells as compared to both the control and *SGPL1*-silenced cells, which coincided with the lowest S1P/Cer ratio and downregulation of Cathepsin-D mRNA expression. Interestingly, *SGPL1* silencing led to downregulation of the Caspase-3 protein and its active form, despite increased Caspase-9 Tyr153 phosphorylation. *SGPL1*-silenced cells also displayed the highest S1P/Cer ratio and high protein expression of Cathepsin D, yet significantly higher cellular death than controls.

A high level of mRNA expression and protein level of Cathepsin-D were observed in colorectal cancer [43]. Up to 78% of CRC cases strongly express Cathepsin-D, while no significant staining is observed in the normal mucosa [68]. Additionally, it was found that Cathepsin-D expression is greatest in invasive areas, suggesting its involvement in tumor progression [68]. Inhibition of Cathepsin-D markedly enhances anticancer drug-induced apoptosis in a few human cancer cells [42]. Cathepsin-D, the most abundant lysosomal protease, appears to have diverse properties, from pro-apoptotic to pro-invasive, and pro-metastatic. This underlines that its effect on cell death is controversial and depends on cellular type, stimulators, and cell context [42,43,69]. Besides, Cathepsin-D mainly exists in lysosomes, but under specific conditions also localizes in the cytosol and extracellular space, displaying involvement in apoptosis and metastasis, respectively. Therefore, the ability to modulate cell death and proliferation may differ also based on Cathepsin-D localization [42].

The direct effects of sphingolipids on Cathepsin-D activity can yield inhibition or activation, which depends on the type and structure of the sphingolipid species. Sphingosine and short-chain ceramide inhibits Cathepsin-D activity, whereas sphingolipid phosphates were shown to activate this enzyme at low micromolar range (in both cases in a pH-dependent manner) [44]. Ceramide-1-phospahte displayed the strongest activatory properties, with S1P being somehow weaker in this aspect. At the gene expression level, modulation of the content of both the S1P and CER affects cathepsins expression. In prostate cancer cells, degradation of ceramide through the action of lysosomal acid ceramidase upregulates Cathepsin-B (CTSB) expression in a S1P-dependent manner [70]. Contrary to the above findings, ablation of the *SGLP1* gene in mouse embryonic fibroblasts inhibits maturation of Cathepsin-D and decreases overall lysosomal activity [71]. Regarding ceramide, its accumulation is crucial in the activation of CTSB in the cellular model of Parkinson’s disease [72]. In light of the above data, we can hypothesize that expression of Cathepsin-D in CRC cells follows overall S1P content and the S1P/Cer ratio, with the lowest mRNA expression in this enzyme concomitant with a low S1P/Cer ratio and S1P content in *SPHK1*-silenced cells and the highest protein expression under a high S1P/Cer ratio and S1P accumulation in *SGLP1*-silenced cells.

In our study, silencing the *SPHK1* gene in HCT-116 cells resulted in changes not only restricted to the expected reduction in S1P content, but also caused an increase in the level of sphingosine and some ceramides (C18:0-Cer and C18:1-Cer). As a result, we observed a significantly reduced S1P/Ceramide ratio, which is critical to cell death and survival. Accordingly, we observed activation of Caspase-3 and an increase in the apoptosis-mediated cell death. Moreover, *SPHK1*-silenced cells displayed an increase in the expression of Cas-9 and Cas-3, and a decrease in expression of Cathepsin-D at the mRNA level.

Sphingosine-1-phosphate lyase located in the endoplasmic reticulum is a membrane-bound important enzyme, which irreversibly hydrolyzes S1P, and acts as the metabolic sink for the highly bioactive sphingoid backbones. In HCT-116 cells with the *SGPL1*-silenced gene, we noted the expected elevation in the content of S1P, but also an increase in the total ceramide content. We also observed that, although the S1P-to-ceramide ratio increased in cells with the silenced *SGPL1* gene, it did not prevent apoptosis. However, in these cells, the levels of ceramide also increased, and it seems that the observed intensification in apoptosis is precisely due to the increase in ceramide levels. The puzzling upregulation of ceramide content in *SGPL1*-silenced cells could be explained by the unique role of the SGLP1 enzyme in sphingolipid metabolism. Contrary to the salvage pathway (sphingosine re-acylation towards complex sphingolipids), SGLP1 ensures permanent degradation of sphingoid backbones of S1P and Spa1P towards phosphoethanolamine and hexadecenal via irreversible reaction. *SGLP1* silencing creates a bottleneck in degradation of S1P and sphingoid backbones located upstream of this enzyme. Contrary to S1P, sphingosine can directly enter the ceramide synthesis pathway, which, in the case of the inhibition of sphingoid backbone degradation via *SGLP1* silencing, can increase ceramide accumulation. This effect was likely the reason for the increase of CER content in the *SGLP1*-silenced cells.

Previous reports showed that SGPL1 was downregulated in colon cancer tissue, leading to S1P accumulation in neoplastic intestinal tissues [28]. A significant reduction in SGPL1 expression and activity was also found in adenomas and colitis-associated carcinoma in the mouse model compared to the control [28,73]. However, in contrast to these findings, the increased SGPL (and SPHK) mRNA levels were recently reported in human colon cancer tissues compared with the adjacent nontumorous tissues [74]. Moreover, loss of SGPL1 promotes oncogenesis [75], and reduction of SGPL levels in HCT 116 cells led to reduced cell proliferation and invasion, but unaltered migration [74]. It seems that the expression of SGPL in colorectal cancer tissue may change depending on the tumor stage, hence the different results of the SGPL role in different patient populations. The variable expression in cancer tissue of enzymes (or genes) involved in the synthesis and degradation of ceramides could also explain the varying levels of ceramides in cancer tissue observed in our previous study [1]. RT-PCR analysis of the surgically resected samples showed that SGPL1 mRNA tended to be highly expressed in human colorectal cancer tissues compared with normal mucosa tissues [76]. SGPL1 mRNA expression was by far the highest among all S1P metabolizing enzymes in two human colorectal cancer cell lines; selective reduction of SGPL1 mRNA expression (SPHK1, SPHK2, SGPP1, and SGPP2 remained unchanged) and complementary reduction of the SGPL1 enzyme level increased intracellular S1P levels but did not affect cell proliferation or metabolic activity of the cells [77]. It has been shown that diminished SGPL1 expression induced a partial redifferentiation of human colorectal cancer cell line towards normal colon epithelial cells, as a result of E-cadherin upregulation, an increase in intercellular adhesion, and inhibited cell migration. It seems that a change in SGPL1 expression is associated with the malignancy of already established colon cancer cells [77]. However, other studies showed a different mechanism of SGPL1 involvement in carcinogenesis [73,76] and revealed that SGPL1 knockout also promoted the transformation of normal mouse colon epithelium into cancer cells and provoked the immediate appearance of neoplastic tumors [78].

## 4. Materials and Methods

### 4.1. Cell Culture Experiments

The human colorectal carcinoma cell line (HCT-116) was purchased from American Type Cell Culture Collection (ATCC). HCT-116 were cultured in dedicated media (McCoy 5A, Gibco, New York, NY, USA) containing 10% fetal bovine serum (FBS, Gibco), streptomycin (50 µg/mL) and penicillin (50 units/mL; Gibco). Additionally, media were supplemented with albumin-conjugated free fatty acids (150 µM), namely, oleic and palmitic in a ratio 2:1 (mol/mol). Cell lines were cultured in humanified conditions (37 °C, 5% CO_2_). Cells from the third to sixth passage were acquired for experiments.

To silence the expression of *SPHK1* and *SGPL1* genes, we employed a siRNA-mediated RNA interference approach [79]. A total of 250 × 10^3^ HCT-116 cells were seeded on a 6-well plate and incubated with esiRNA (10 nM/L) and 10 µL of transfectant per well (both supplied by Sigma Aldrich, Saint Louis, MO, USA). *SPHK1 (HCT(SPHK1-))* and *SGPL1 (HCT(SGPL1-))* genes were silenced, while human eGFP was used as control (HCT(CON)). After 72 h from transfection, cells were collected for Western blot, qPCR assay, and lipidomics. All the experiments were repeated four times (n = 4), except qPCR assays (n = 6).

### 4.2. Evaluation of Cellular Apoptosis

Cell culture was established as described above. Supernatants were collected for ELISA detection of histone release in apoptotic bodies. The ELISA procedure (Cell Death Detection ELISA Plus, Roche, Indianapolis, IN, USA) was performed according to standard manufacturer protocol. Results were acquired using the VarioscanLux instrument (ThermoFisher, Waltham, MA, USA) at 490 nm.

### 4.3. Sphingolipid Measurements

The level of sphingolipids was measured by the UHPLC/MS/MS method, according to Blachnio-Zabielska et al. [80], with minor modifications. Briefly, cells were homogenized in a buffer consisting of 0.25 M sucrose, 25 mM KCl, 50 mM Tris, and 0.5 mM EDTA, pH 7.4. The protein concentration in each homogenate was determined using a Pierce660nm protein assay kit (Thermo Fisher Scientific). Immediately afterwards, a mixture of internal standards (Sph-d7, SPA-d7, S1P-d7, C15:0-d7-Cer, C16:0-d7-Cer, C18:1-d7-Cer, C18:0-d7-Cer, 17C/20:0-Cer, C24:1-d7-Cer, C24-d7-Cer Avanti Polar Lipids, Alabaster, AL, USA) was added to each sample and the samples were vortexed. After that, an extraction mixture (isopropanol:water:ethyl acetate, 30:10:60; *v:v:v*) was added to each sample, followed by sonication and centrifugation at 4000 rpm for 10 min at 4 °C. After extraction, the samples were dried under a stream of nitrogen and suspended in LC Solvent B (2 mM ammonium formate, 0.1% formic acid in methanol) for UHPLC/MS/MS analysis. The chromatographic separation was performed on a reversed-phase column—Zorbax SB-C8 2.1 × 150 mm, 1.8 μm (Agilent Technologies, Santa Clara, CA, USA) in a binary gradient, using 1 mM ammonium formate, 0.1% formic acid in water as solvent A, and 2 mM ammonium formate, 0.1% formic acid in methanol as solvent B, at the flow rate of 0.4 mL/min. Sphingolipids were analyzed with the use of a Sciex QTRAP 6500 + triple quadrupole mass spectrometer (AB Sciex Germany GmbH, Darmstadt, Germany) with multiple reaction monitoring (MRM) against standard curves constructed for each compound.

### 4.4. Western Blotting

Proteins were isolated from cells in RIPA buffer (Sigma-Aldrich) containing 0.5 mM TCEP (Sigma-Aldrich) and protease and phosphatase inhibitors (cOmplete ULTRA mini and PhosSTOP tablets, Roche). The content of protein in homogenates was measured using a Pierce 660nm protein assay kit (Thermo Fisher Scientific). Fatty-acid-free bovine serum albumin was used as a standard. After denaturation in Laemmli buffer, proteins (30 µg) were separated by SDS-PAGE (Criterion Cell electrophoresis cell and Criterion TGX midi Any kD gel) and transferred to a PVDF membrane (BioRad Trans Blot SD semi-dry transfer cell with a discontinuous Tris/CAPS buffer system; 15% methanol for the anode and Tris/CAPS/0.1% SDS for the cathode). Membranes were incubated with the appropriate primary antibody. The following target proteins were quantified using primary antibodies: sphingosine kinase 1 (SphK1), sphingosine-1-phosphate lyase 1 (SGPL1), Caspase-3, active Caspase-3 (cleaved), Caspase-9, Caspase-9 (Tyr-153), Cathepsin-D, and vinculin (vendor Cell Signaling Technology, Danvers, MA, USA). To detect primary antibody binding to protein, the membrane was incubated with an HRP-conjugated secondary antibody, followed by incubation in a Clarity ™ Western ECL chemiluminescent substrate (Bio-Rad) and visualization using a Bio-Rad ChemiDoc XRS + imaging system. Band intensities were quantified with the Bio-Rad Image Lab software package. The obtained values were normalized to the vinculin protein expression, as measured from parallel runs and expressed as fold changes over control group value. Unless otherwise stated, all chemicals and equipment used for immunoblotting were purchased from Bio-Rad (Hercules, CA, USA). Unless stated otherwise, all antibodies were purchased from Thermo Fisher Scientific (Waltham, MA, USA).

### 4.5. Real-Time PCR

Total RNA was isolated from cells with the use of a mirVana Isolation Kit (ThermoScientific, USA) according to the manufacturer’s instructions. The RNA was reverse-transcribed into cDNA using a Transcriptor First Strand cDNA Synthesis Kit (Roche). Real-time PCR was performed with the use of the following primers: sphingosine kinase 1 (SphK1), forward, 5′-TTCCTTGAACCATTATGCTG-3′, reverse, 5′-GATACTTCTCACTCTCTAGGTC-3′; Sphingosine-1-phosphate lyase 1 (SGPL1), forward, 5′-AATGAGAAGAGCTATCTCCAG-3′, reverse, 5′-TTTTGTATTTGACAGCCAGC-3′; Caspase-3, forward, 5′-TTTCGTGAGTGCTCGCAGC-3′, reverse, 5′-CCTTTATTAACGAAAACCAGAGCG-3′; Caspase-9, forward, 5′-TTGGTTCTGGAGGATTTGGT-3′, reverse, 5′-TGCTCAGGATGTAAGCCAAA-3′; Cathepsin-D, forward, 5′-GCTGATTCAGGGCGAGTACA-3′, reverse, 5′-TCCCAGCTTCAGTGTGATCG-3′ and glyceraldehyde 3-phosphate dehydrogenase (GAPDH), forward, 5′-TCGGAGTCAACGGATTTG-3′, reverse, 5′-CAACAATATCCACTTTACCAGAG-3′. GAPDH was used as a housekeeping gene. PCR reaction was performed using a LightCycler480 system (Roche Mannheim, Germany). The results were normalized to GAPDH expression measured in each sample and the relative expression levels were evaluated using the ΔΔCt method.

### 4.6. Statistical Analyses

GraphPad Prism 9 was used to perform all statistical analyses. The results were expressed as median from 4 independent experiments (n = 4; except qPCR n = 6) with an interquartile range. Significant differences were identified by the nonparametric Mann–Whitney test. The significance threshold was set at *p* < 0.05.

## 5. Conclusions

Our findings confirmed that the modification of sphingolipid metabolism at the level of sphingosine-1-phosphate and the S1P/Cer ratio through silencing of *SPHK1* or *SGPL1* affects the apoptosis of CRC cells. The decrease in S1P levels and the S1P/Cer ratio at the same time results in a significant increase in CRC cell apoptosis, which was accompanied by upregulation in the active form of executioner Caspase-3. Conversely, an increase in S1P levels and the S1P/Cer ratio in *SGPL1*-silenced cells led to downregulation of Caspase-3 active form and a subsequent increase in Cathepsin-D protein expression in CRC cells. The downregulation of active Caspase-3 and upregulation of Cathepsin-D suggest increased pro-metastatic capacity of those cells.

## Figures and Tables

**Table 1:** Composition of individual Cer molecular species in *SPHK1*- and *SGPL1*-gene-silenced human colorectal tumor HCT-116 cells.

	HCT_(CON)_			HCT_(SPHK1-)_			HCT_(SGPL1-)_		
Ceramide	Q1	Median	Q3	Q1	Median	Q3	Q1	Median	Q3
C14:0	22.25	23.56	24.9	22.46	24.55	25.15	20.71	21.45	22.92
C16:0	419.2	462.6	489.7	393.3	426.6	436.5	547.8	566.8 *	594.2
C18:1	10.51	10.78	11.08	11.9	12.75 *	13.03	13.21	13.99 *	14.29
C18:0	8.59	9.09	10.56	11.75	12.29 *	14.2	14.76	16.14 *	16.55
C20:0	3.64	3.83	4.46	3.57	3.68	4.14	4.93	5.25 *	5.43
C22:0	19.99	23.16	24.14	24.38	26.38	27.29	30.08	31.05 *	33.51
C24:1	402.2	409.5	412.5	391.5	407.9	439.3	409.9	421.4	450.9
C24:0	76.89	81.08	86.43	78.98	82.76	87.99	84.01	87.87	89.05

Values are expressed as median with an interquartile range (Q1; Q3) (n = 4). * *p* < 0.05 vs. control (HCT_(CON)_).

## Data Availability

Not applicable.
